# Scarless Laparoscopic Cholecystectomy Combined With Lipoabdominoplasty

**DOI:** 10.7759/cureus.64561

**Published:** 2024-07-15

**Authors:** Dimitra Daskalopoulou, Nikolaos Moustakis, Christos Barkolias

**Affiliations:** 1 Department of Anatomy, National and Kapodistrian University of Athens, Athens, GRC; 2 Department of Plastic and Reconstructive Surgery, Naval Hospital of Athens, Athens, GRC; 3 Department of General Surgery, Naval Hospital of Athens, Athens, GRC

**Keywords:** laparoscopic surgery, minimally invasive surgery, plastic surgery, scarless, laparoscopic cholecystectomy, lipoabdominoplasty

## Abstract

As the aesthetic expectations of our society are rising, the patients are increasingly inquiring about ways to reduce the postoperative scars and avoid multiple operations. Herein, we present a case of cholecystectomy combined with lipoabdominoplasty whereby gallstone disease, abdominal wall laxity and skin excess were concurrently addressed, thereby eliminating the need for trocar-incisions on the abdominal skin. We can conclude that lipoabdominoplasty with laparoscopic cholecystectomy is a safe combined procedure that can be performed selectively in patients with cholecystolithiasis and body contour problems leading to improved cosmetic results without the presence of unnecessary scars.

## Introduction

The aesthetic result following a surgical procedure is a common concern among patients, who are developing higher expectations more and more. Combined surgery addressing multiple problems is an attractive option reducing operative and anesthesia sessions, hospital stay and the overall cost [1]. Abdominoplasty is nowadays one of the most widely demanded and performed surgical procedures concerning deformities from musculofascial laxity as well as excess skin and fat tissue and it is increasingly performed concomitant with other aesthetic procedures [2]. We present a case of simultaneous implementation of abdominal body contour surgery with laparoscopic cholecystectomy with the aim of achieving safely better cosmetic results avoiding unnecessary postoperative scars.

## Case presentation

**Figure 1:**
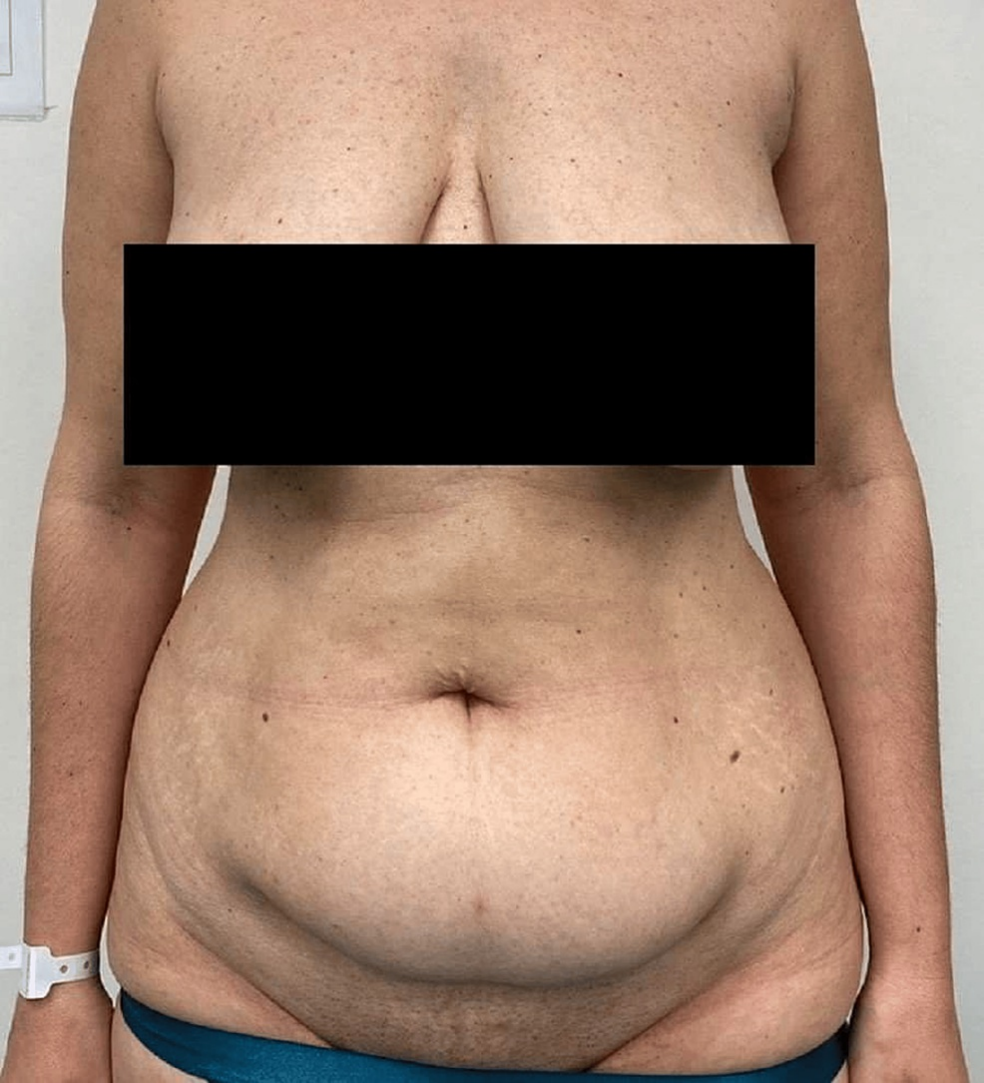
Preoperative image of the patient

A 42-year-old woman presented to our hospital due to recurrent biliary colic and recurrent episodes of abdominal pain in the right upper quadrant. The abdominal ultrasound examination revealed multiple gallbladder stones without any signs of acute inflammation or distention of the biliary tract. Besides the biliary colic, the patient displayed abdominal wall laxity and skin excess after weight loss of 20 kg and multiparity, hence, expressing the desire of treating the abdominal wall as well (Figure 1).

**Figure 2:**
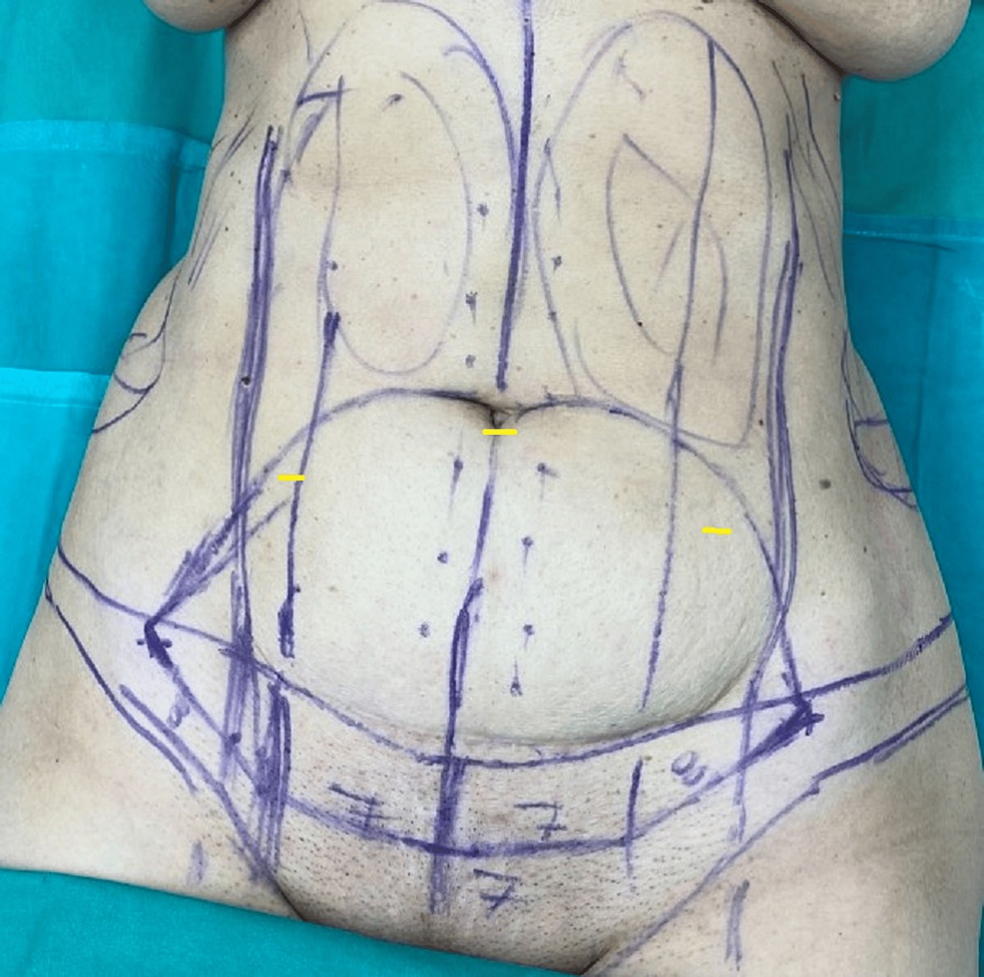
Preoperative image of the markings of the abdominoplasty incision and sites of liposuction Yellow lines show the positions of the incisions for the liposuction.

The surgical treatment comprised three stages: the liposuction and preparation of the abdominal flap followed by the laparoscopic cholecystectomy and, finally, the treatment of the rectus muscle diastasis and wound closure. The procedure was initiated in supine position under general anesthesia and following administration of 1 L of superwet local anesthesia (1 L Ringer’s lactate, 40 mL ropivacaine 7.5 mg/mL, 1 mL 1:1000 epinephrine). Three incisions were made, with one on the lower pole of the umbilicus and two on both sides of the marked ellipse of a standard abdominoplasty incision on the abdominal skin (Figure 2). Liposuction was then performed on the upper abdominal region up to the coastal lines and xiphoid process, as well as on the lower abdominal wall to facilitate the dissection of the abdominal flap. The procedure of a standard abdominoplasty was initiated by releasing the umbilicus circumferentially and incising the lower limb of the ellipse. The upper abdominal flap was undermined respecting the lateral blood supply of the abdominal wall. Additionally, a subcutaneous tunnel was created up to the xiphoid process.

**Figure 3:**
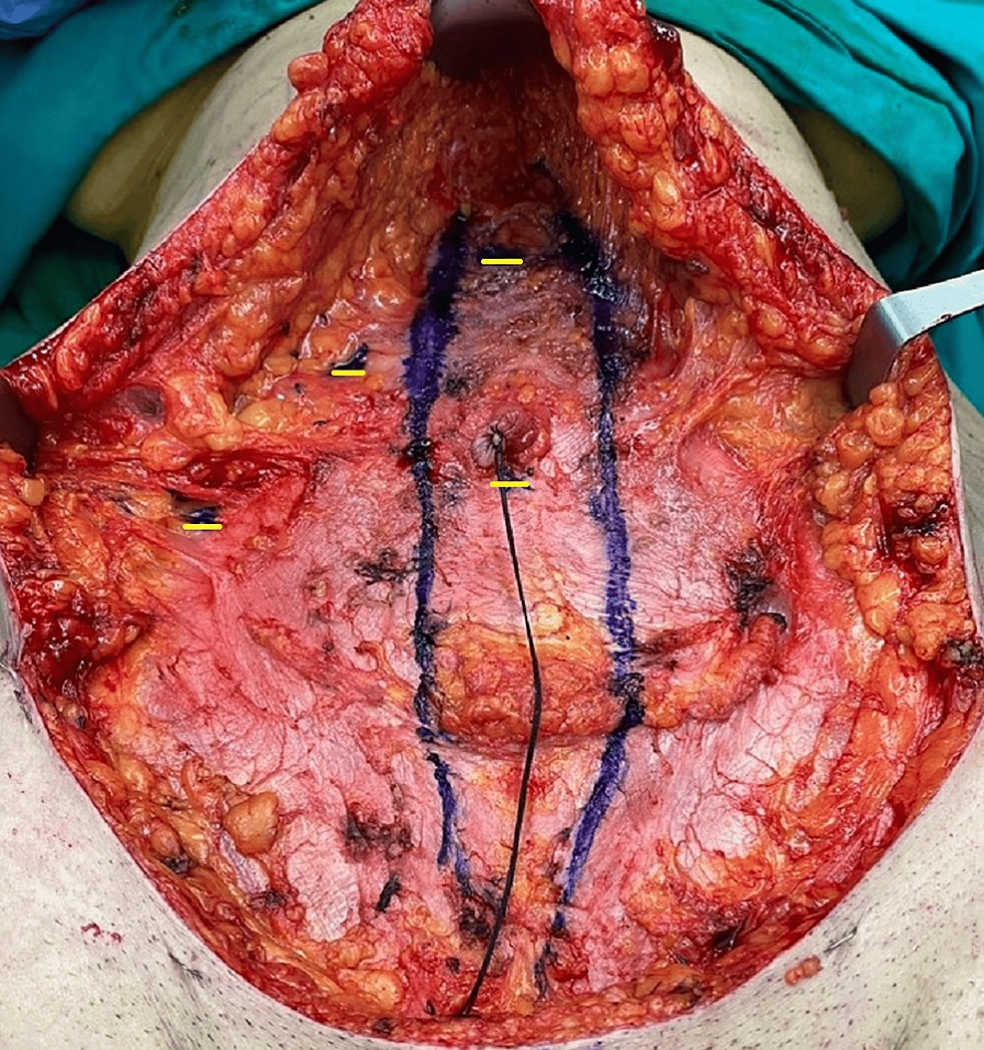
Intraoperative image of the abdominal wall before inserting the trocars, with the rectus muscle diastasis also marked Yellow lines show the positions of the four trocar incisions on the abdominal wall.

**Figure 4:**
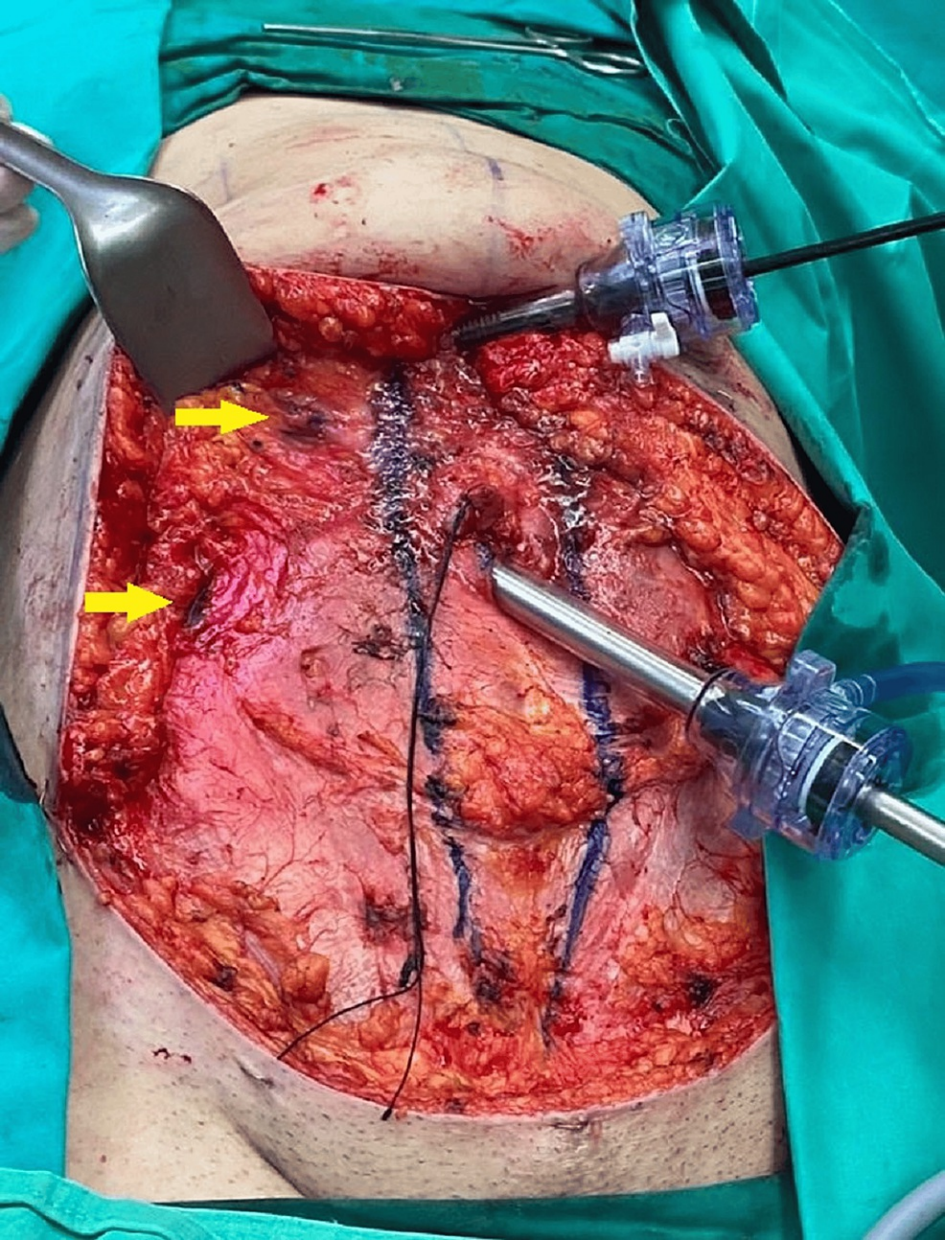
The position of trocars on the abdominal wall following retraction of the abdominal flap The rectus muscle diastasis was marked before performing an anterior sheath plication. Yellow arrows show the positions where each of the 5-mm trocars is inserted.

**Figure 5:**
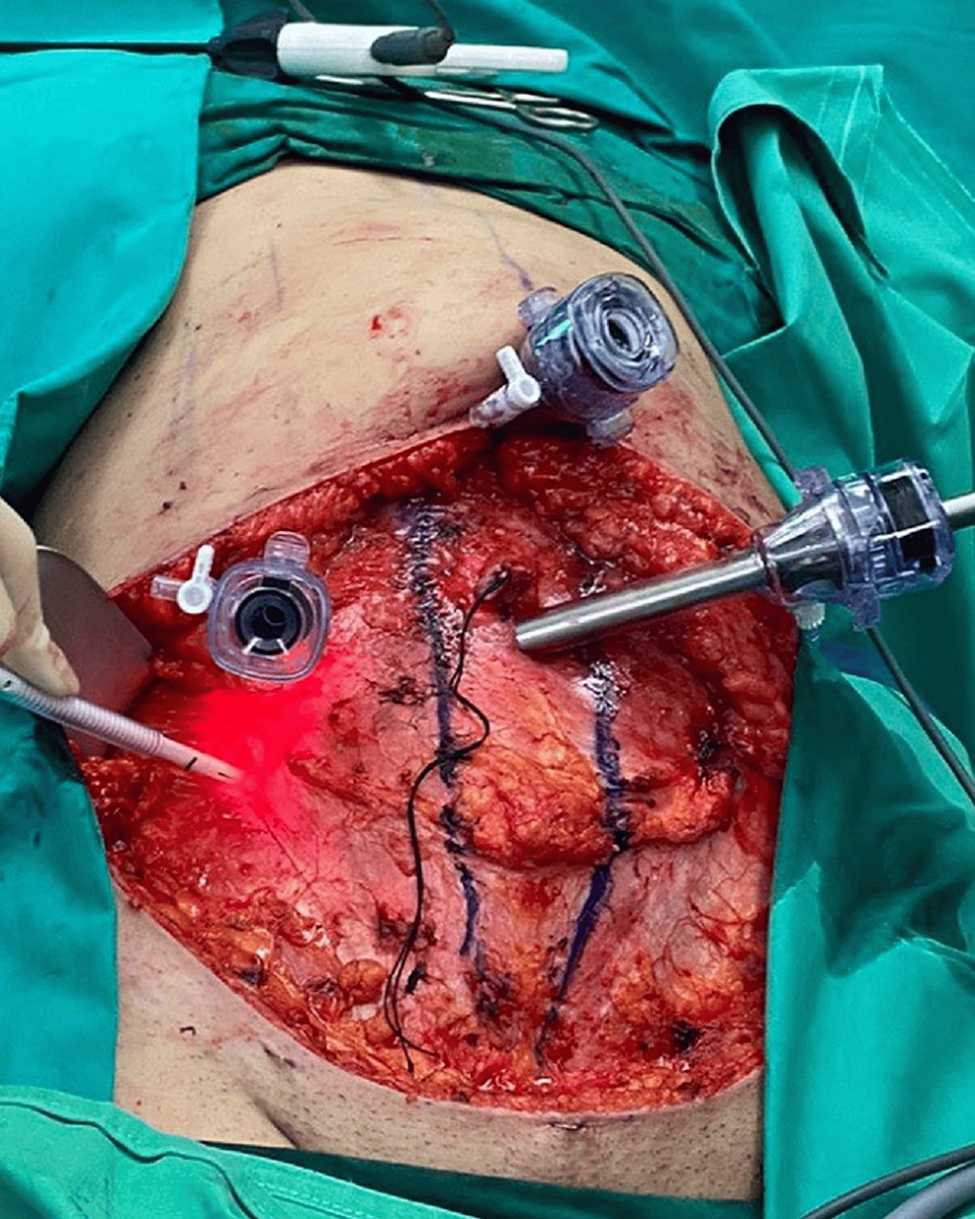
The 5-mm trocars inserted under direct view on the right abdominal wall

After meticulous hemostasis, the second stage of the surgical treatment was initiated by retracting the abdominal flap and inserting a 10-mm Hasson trocar inferiorly to the umbilicus and directly on the fascia and muscle to generate pneumoperitoneum at 12 mm Hg (Figure 3). A typical laparoscopic cholecystectomy was performed by introducing one 10-mm trocar inferiorly to the xiphoid process, and two 5-mm trocars on the right lateral epigastric wall under direct view (Figures 4-5). After performing Critical View of Safety, the cystic artery and the cystic duct were identified, properly dissected and clamped. The gallbladder was removed through the umbilical port using a retrieval bag and the abdominal wall incisions were closed with PDS 2-0 stiches.

**Figure 6:**
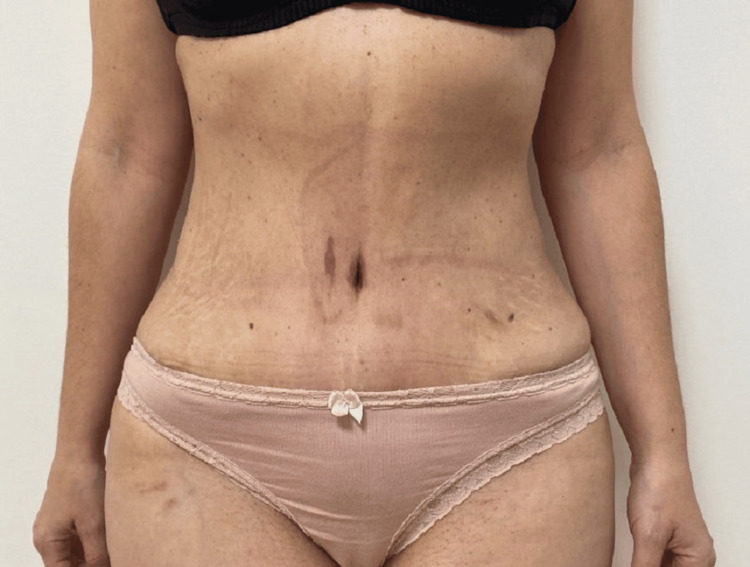
Postoperative image of the patient six months after the procedure

During the final segment of the procedure, the rectus muscle diastasis was repaired performing an anterior sheath plication using non-absorbable sutures. Dermolipectomy of the infra-umbilical region was conducted after flexing the operating room table and the umbilicus was exteriorized in the new position on the abdominal flap. Finally, the abdominoplasty wound was closed in layers with two suction drains left in situ in the plane of dissection. The final result revealed no signs of the cholecystectomy procedure leading to an improved aesthetic outcome. The postoperative recovery was uneventful, and the patient was discharged on postoperative day 1. Paracetamol was the sole medication used for postoperative pain management. During the follow-up consultation, the patient expressed satisfaction with the overall result and procedure (Figure 6).

## Discussion

Gallstone disease is a common pathological entity affecting approximately 20% of the adult population. Gender, age, body mass index and the prevalence of metabolic syndrome constitute factors that strongly correlate with the development of gallstones [3]. Cholecystectomy is the milestone in the treatment of symptomatic cholecystopathy, as well as in the prevention not only of common but also rare complications such as gallstone ileus [4]. Traditionally, the laparoscopic cholecystectomy is conducted through four trocar incisions on the abdominal wall [5]. The use of natural orifices (natural orifice transluminal endoscopic cholecystectomy, or NOTEC) as well as single transumbilical ports (single-incision laparoscopic cholecystectomy, or SILC) has been described with the intention of achieving better cosmesis and less postoperative pain [5-7]. Needlescopic cholecystectomy, i.e., the removal of the gallbladder using instruments with smaller diameters, has also been introduced as an alternative method for more refined aesthetic results in minimally invasive surgery. However, these techniques are accompanied by technical challenges and extensive learning curves [5,6].

Abdominoplasty is one of the most commonly requested cosmetic surgical procedures addressing the anterior abdominal musculo-fascial deficit, redundant skin and fat [2,8]. Performing additional procedures in conjunction with abdominoplasty has become a common practice due to the decrease in surgical and anesthetic sessions and, also, the overall recovery time and cost [1]. The spectrum of combined surgeries includes colostomy revisions, ventral hernia repairs and gynecological operations as well as aesthetic operations such as breast reduction and mastopexy [2]. Various studies have explored the morbidity rates that arise following such combined surgeries supporting the sustenance of safety as well as patient satisfaction [1,2,9,10]. Winocour et al., however, analysed a large cohort of patients who underwent abdominoplasty with additional procedures suggesting that individual risk factors should be rigorously evaluated due to the potential of higher complication rates [11].

In 2001, Saldanha et al. developed lipoabdominoplasty as a means to preserve the abdominal perforating vessels and limit the size of dead space on the lateral abdominal wall compared to standard abdominoplasty [12]. A recent meta-analysis showed that lipoabdominoplasty is associated with lower morbidity and complications than traditional abdominoplasty [13].

In this case, the benefits of lipoabdominoplasty were compounded with the laparoscopic treatment of cholecystolithiasis eliminating the cutaneous trocar incisions. Cases of simultaneous laparoscopic cholecystectomy with traditional abdominoplasty have been reported advocating an overall ameliorated cosmetic result without compromising the safety of the patient [13-17]. In our case, the laparoscopic removal of the gallbladder was successfully and uneventfully conducted under the abdominal skin following sculpture of the abdominal contour using liposuction and abdominoplasty granting an even more aesthetically refined outcome.

## Conclusions

The present case study demonstrates the efficacy of combining lipoabdominoplasty with laparoscopic cholecystectomy in a single procedure, eliminating, thus, adjunct surgical interventions and reducing the overall operative and recovery time, as well as cost and resources. A classic laparoscopic cholecystectomy can be safely conducted in cases of gallstone disease with concomitant presence of abdominal panniculus through the standard abdominoplasty incision, avoiding in this manner any additional cutaneous trocar incisions. The principles of abdominal contour surgery can be safely applied without obstructing the sequence of steps followed during a minimally invasive cholecystectomy granting a superior aesthetic outcome compared to the one achieved following independent surgical procedures. However, proper contemplation of patient criteria is suggested as an inflammation of the gallbladder could potentially increase the risk of postoperative sequelae such as infection and impaired wound healing, posing, accordingly, the need for relevant studies to further support the safety of such combined procedures under different conditions.

## References

[1] Sinno S, Shah S, Kenton K, Brubaker L, Angelats J, Vandevender D, Cimino V (2011). Assessing the safety and efficacy of combined abdominoplasty and gynecologic surgery. Ann Plast Surg.

[2] Simon S, Thaller SR, Nathan N (2006). Abdominoplasty combined with additional surgery: a safety issue. Aesthet Surg J.

[3] Sun H, Warren J, Yip J, Ji Y, Hao S, Han W, Ding Y (2022). Factors influencing gallstone formation: a review of the literature. Biomolecules.

[4] Mulita F, Tchabashvili L, Bousis D (2021). Gallstone ileus: a rare cause of small intestine obstruction. Clin Case Rep.

[5] Zhao JJ, Syn NL, Chong C (2021). Comparative outcomes of needlescopic, single-incision laparoscopic, standard laparoscopic, mini-laparotomy, and open cholecystectomy: a systematic review and network meta-analysis of 96 randomized controlled trials with 11,083 patients. Surgery.

[6] Evers L, Bouvy N, Branje D, Peeters A (2017). Single-incision laparoscopic cholecystectomy versus conventional four-port laparoscopic cholecystectomy: a systematic review and meta-analysis. Surg Endosc.

[7] Zornig C, Mofid H, Emmermann A, Alm M, von Waldenfels HA, Felixmüller C (2008). Scarless cholecystectomy with combined transvaginal and transumbilical approach in a series of 20 patients. Surg Endosc.

[8] Matarasso A, Matarasso DM, Matarasso EJ (2014). Abdominoplasty: classic principles and technique. Clin Plast Surg.

[9] Kryger ZB, Dumanian GA, Howard MA (2007). Safety issues in combined gynecologic and plastic surgical procedures. Int J Gynaecol Obstet.

[10] Gemperli R, Neves RI, Tuma P Jr, Bonamichi GT, Ferreira MC, Manders EK (1992). Abdominoplasty combined with other intraabdominal procedures. Ann Plast Surg.

[11] Winocour J, Gupta V, Ramirez JR, Shack RB, Grotting JC, Higdon KK (2015). Abdominoplasty: risk factors, complication rates, and safety of combined procedures. Plast Reconstr Surg.

[12] Saldanha OR, Azevedo SF, Delboni PS, Saldanha Filho OR, Saldanha CB, Uribe LH (2010). Lipoabdominoplasty: the Saldanha technique. Clin Plast Surg.

[13] Xia Y, Zhao J, Cao DS (2019). Safety of lipoabdominoplasty versus abdominoplasty: a systematic review and meta-analysis. Aesthetic Plast Surg.

[14] Gençtürk M, Erdem H, Sözen S (2021). Simultaneous scar-less laparoscopic cholecystectomy with abdominoplasty. Arch Clin Exp Med.

[15] Prasad MB, Surapaneni SR, Dabade SS (2012). Scarless cholecystectomy: laparoscopic cholecystectomy with abdominoplasty. Indian J Surg.

[16] Ibrahim AAM (2017). Simultaneous laparoscopic cholecystectomy with abdominoplasty. Int Surg J.

[17] Özgur F, Aksu A, Özkan O, Hamaloglu E (2002). The advantages of simultaneous abdominoplasty, laparoscopic cholecystectomy and incisional hernia repair. European J Plastic Surg.

